# Ecological influences on host–parasite dynamics among *Biomphalaria* snails in two schistosomiasis endemic regions of Kenya

**DOI:** 10.3389/fpara.2026.1729760

**Published:** 2026-02-10

**Authors:** Florence N. Parsimei, Steven Ger Nyanjom, Mercy Y. Akinyi, George Ogara, Collins Ngudi, Patrick K. Karanja, Maurice R. Odiere, Lucy Ochola

**Affiliations:** 1 Department of Biochemistry, Jomo Kenyatta University of Agriculture and Technology, Nairobi, Kenya; 2 Tropical Infectious Diseases Department, Kenya Institute of Primate Research, Nairobi, Kenya; 3 Neglected Tropical Diseases Unit, Kenya Medical Research Institute- Center for Global Health Research, Kisumu, Kenya

**Keywords:** *Biomphalaria* snails, environmental factors, host–parasite dynamics, Lake Victoria, Mwea Irrigation Scheme, trematodes

## Abstract

Schistosomiasis is a neglected tropical disease affecting over 240 million people globally, with sub-Saharan Africa bearing the highest burden. In Kenya, transmission of *Schistosoma mansoni*, the causative agent of intestinal schistosomiasis, remains prevalent in western, coastal, and central regions, particularly in the Mwea Irrigation Scheme and the Lake Victoria basin. The parasite depends on *Biomphalaria* snails as intermediate hosts, yet ecological determinants influencing infection dynamics remain underexplored. This study examined *S. mansoni* infection in *Biomphalaria* snails across two contrasting ecosystems: Lake Victoria and the Mwea rice irrigation scheme in Kenya. Snails, water, and soil samples were collected from the study sites. Water and soil were analyzed for abiotic parameters, including temperature, turbidity, salinity, pH, and soil porosity, while snail infections were confirmed via cercarial shedding and PCR targeting the ITS region. Laboratory-maintained isolates of *S. mansoni* were passed through baboons and served as positive controls for molecular identification. *Biomphalaria pfeifferi* was the most dominant species (90.4% of all snails sampled). Infection prevalence among infected snails varied across sites around Lake Victoria basin: Anyanga Beach, Siaya (70.8%, 80/113), Sindo Rangwena, Homabay (20.6%, 7/34), Kasabong, Siaya (16.9%, 12/71), and Kendu Bay, Homabay (16.7%, 3/18), with a chi-squared test confirming a strong site–infection association (χ² = 67.33, df = 3, p < 0.001), indicating significant spatial heterogeneity in transmission risk. Infection correlated positively with temperature (r = 0.72, p < 0.01) and soil porosity (r = 0.65, p < 0.05), and negatively with turbidity (r = −0.63, p < 0.01) and salinity (r = −0.58, p < 0.05) for samples found in areas around Lake Victoria basin. Molecular screening of 272 snail-derived samples using ITS1 primers yielded 113 positives. Sequencing confirmed *B. pfeifferi* (600 bp) from Mwea Irrigation Scheme and Lake Victoria Basin, forming a monophyletic clade with strong bootstrap support. The 500 bp ITS1 fragment identified *S. mansoni* in lab-maintained strains and Thiba in Mwea Irrigation Scheme samples, clustering within the *S. mansoni* clade. Further analysis of cercariae using 18S rDNA revealed ≥98% similarity to *Zygocotyle lunata* in Lake Victoria sites, forming a well-supported clade distinct from schistosomes.

## 1 Introduction

Schistosomiasis, a parasitic disease caused by *Schistosoma* species, remains one of the most neglected tropical illnesses, predominantly affecting populations in sub-Saharan Africa. An estimated 240 million individuals are infected globally, with over 90% of the burden concentrated in sub-Saharan Africa (Hotez et al., 2019; World Health Organization, 2022). The disease thrives in communities lacking clean water and sanitation, resulting in significant morbidity and mortality, with the World Health Organization (WHO) estimating over 200,000 deaths annually (WHO, 2023). In Kenya, approximately 17.4 million people are at risk, with high prevalence rates observed in the Lake Victoria basin, coastal, central, and lower eastern regions (WHO, 2013; Vale et al., 2017). Mwea Irrigation Scheme in Kirinyaga County and areas surrounding Lake Victoria remain particularly hyperendemic (Odiere et al., 2012; Gichuki et al., 2019; Handzel et al., 2003; Steinmann et al., 2006; Steinauer et al., 2008).

The disease presents in intestinal and urogenital forms, with *Schistosoma mansoni* primarily causing intestinal schistosomiasis. The current WHO control strategy involves mass drug administration (MDA) with praziquantel and snail control wherever feasible. However, MDAs fail to eliminate juvenile worms and do not prevent reinfection. Malacological surveys for *schistosome* infection detection are always not feasible in the surveillance of schistosomiasis.

*Biomphalaria* snails, the intermediate hosts of *S. mansoni*, thrive in freshwater bodies, with their abundance influenced by various climatic and ecological factors (Monde et al., 2016). However, the relationship between their infection rates and environmental factors is not fully elucidated. Research indicates that behavioral and environmental factors such as water quality significantly impact snail abundance and infectivity (Mereta et al., 2019).

Traditional methods for cercarial shedding often fail to identify prepatent infections, highlighting the need for more sensitive molecular techniques for effective surveillance (Joof et al., 2020; Hamburger, 2017; Nwoko et al., 2021). This study therefore aims to bridge these knowledge gaps and support the development of localized vector control and disease elimination strategies. Molecular screening is crucial for detection of trematodes that infect *Biomphalaria* snails, as morphology often does not effectively differentiate between closely related species. Blasco-Costa et al. (2016) emphasized the importance of optimal methods for trematode classification, recommending ribosomal and mitochondrial markers such as ITS and COI for accurate precise species determination. demonstrated this approach by sequencing larval trematodes from South African snails, showing that molecular data can clarify phylogenetic placement where morphology is ambiguous. Salloum et al. (2023) also utilized ITS rDNA markers to identify trematode communities in African freshwater snails, uncovering co-infections and their evolutionary relationships. DeJong et al. (2001) and Zhang et al. (2019) provided phylogenetic evidence of African origins of *Biomphalaria* hosts and variability in markers, aiding in precise host identification in endemic areas. These research efforts highlight the significance of molecular methods for species-specific identification and phylogenetic investigation, essential for mapping transmission routes and informing schistosomiasis monitoring. Our study utilizes sequence-based markers (ITS1 and 18S rDNA) and phylogenetic techniques to evaluate relationships among *S. mansoni* and other trematodes infecting *Biomphalaria* snails in Mwea Irrigation Scheme and areas around Lake Victoria basin in Kenya, while additionally investigating environmental factors influencing infection dynamics in the Lake Victoria region.

The objectives were to (1) investigate the influence of environmental factors (water temperature, water turbidity, water pH, soil porosity, and water salinity) on *Biomphalaria* snail infection dynamics around the Lake Victoria basin and (2) to assess the phylogenetic relationships between *S. mansoni* and other trematode species infecting *Biomphalaria* snails in areas around Lake Victoria basin and Mwea Irrigation Scheme. By employing sequence-based markers and phylogenetic methods, this study provides insights into species-level characterization and evolutionary relationships among trematodes infecting shared *Biomphalaria* snail hosts, thereby enhancing understanding of parasite–host associations in endemic freshwater ecosystems in Kenya. In addition, understanding the role of environmental factors in shaping *Biomphalaria* infection dynamics is essential to improve and inform schistosomiasis control efforts in areas around the Lake Victoria basin.

## 2 Materials and methods

### 2.1 Study sites

Fieldwork was conducted between July and October 2023 in two sites in Kenya, Mwea Irrigation Scheme, located in Kirinyaga county and areas surrounding Lake Victoria that encompasses several counties including Homabay, Siaya, Kisumu and Nyamira Counties, which are approximately 470 km apart. Mwea Irrigation Scheme (513 km²) receives 1,200–1,600 mm of annual rainfall Masaku et al. (2015) and contains a large rice irrigation scheme supported by Thiba and Nyamindi Rivers. The region is characterized by flat terrain with black cotton and red soils and is hyperendemic for *S. mansoni* (Kihara et al., 2007).

Lake Victoria, Africa’s largest freshwater lake, lies at 1,133 meters above sea level and experiences a tropical climate with temperatures ranging from 21°C to 26°C. The Lake is replenished by direct rainfall and flows from over 20 rivers originating in five East African countries. While the Kagera and Mara are the only transboundary rivers, others such as the Nzoia, Yala, Nyando, Sondu Miriu, and Sio flow from Kenya. Additionally, rivers from Tanzania and Uganda contribute to the lake, which drains solely through the White Nile. Lake Victoria harbors *Biomphalaria* snails which are pivotal in disease transmission. The lake also supports the livelihoods of millions by providing water for farming, fishing, and domestic use (Vanderkelen et al., 2018).

**Figure 1:**
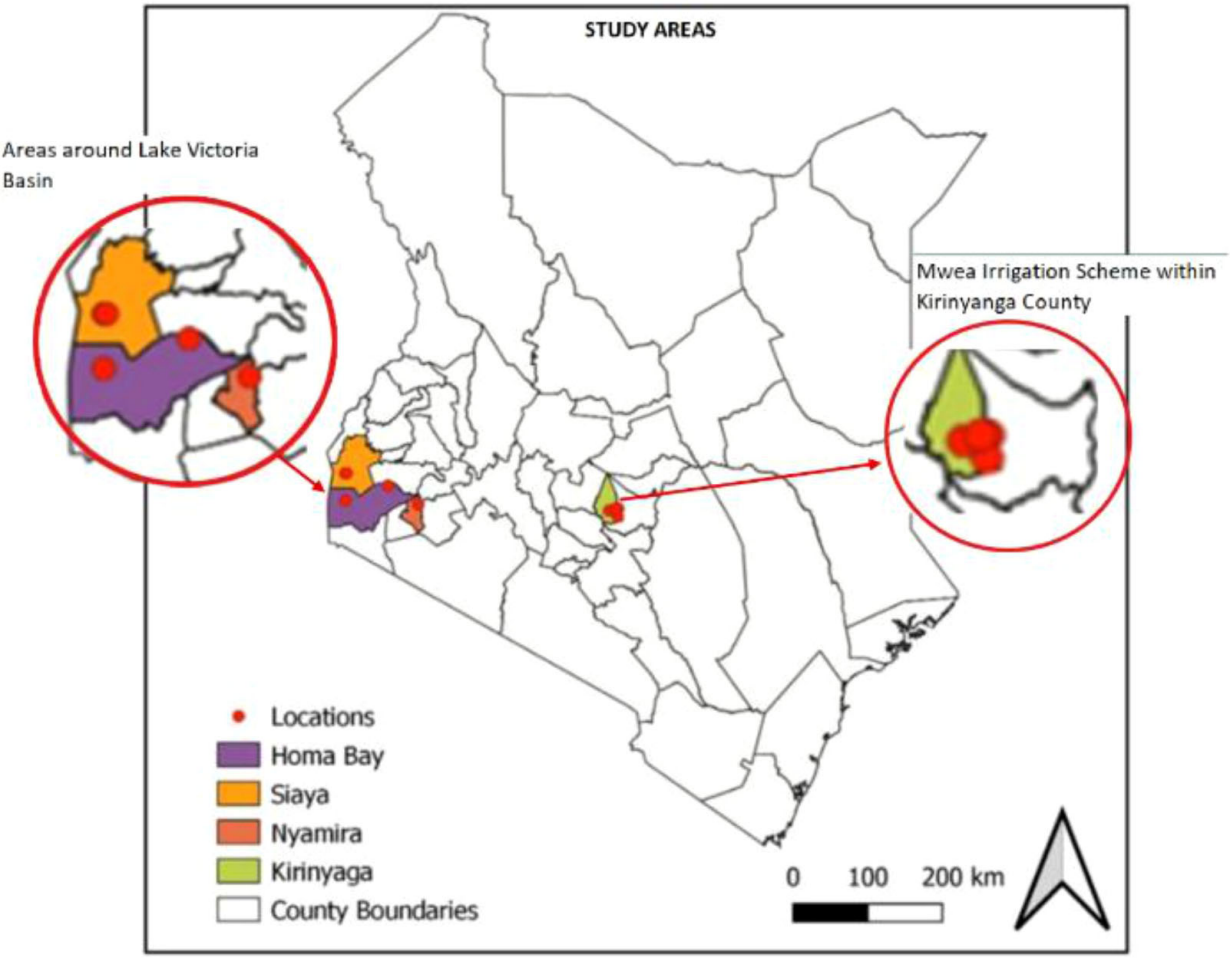
Indicates the study sites in Kenya where sampling was conducted in two geographical zones: Lake Victoria basin (Homa Bay, Siaya, Nyamira) and Mwea Irrigation Scheme (Kirinyaga County). County boundaries are shown in grey; red dots indicate sampling locations.

To ensure appropriate site selection, an initial survey was conducted prior to sampling in both Lake Victoria and Mwea Irrigation Scheme. This survey aimed to identify the presence and distribution of *Biomphalaria* snails, considering that climatic conditions and human activities, such as farming and livestock rearing, can significantly impact snail populations. Specific sampling sites were selected based on direct field observations indicating moderate to high snail abundance during the preliminary assessment, ensuring that sampling was both targeted and ecologically relevant. Sampling sites included Kianganga, Murinduko, Thiba, and Togonye in Mwea Irrigation Scheme (Kirinyaga County), and Anyanga, Kasabong (Siaya County), Rangwena (Homabay County), Asao, and Awach rivers (Kisumu County). GPS coordinates were recorded using Garmin etrex 10 (**Figure 1**).

### 2.2 Water and soil sample collection

Corresponding water and soil samples were collected at each snail sampling site. Water (500 ml) was collected in plastic bottles, pre-rinsed with river water, and sealed. Soil (500 g) was collected by digging to a depth of 15 cm. Samples were stored in cool boxes maintained with ice packs at 4°C and transported to the Kenya Marine and Fisheries Research Institute (KEMFRI), Kisumu for analysis.

### 2.3 Environmental parameter analysis

Temperature was measured *in situ* at each sampling site using a standard mercury−in−glass laboratory thermometer (Gilson Co., USA), with a measurement range of –20 to 110°C, while all other physicochemical parameters were analyzed in the laboratory. Turbidity was assessed using a Lovibond TB350 IR turbidity meter (Germany); pH was measured with a Thermo Scientific Eutech pH electrode (Singapore); and salinity was determined using a HI2003 edge conductivity meter (Italy). Water appearance was recorded visually at the time of sampling. Soil porosity was measured using the saturation method (Moret-Fernández and López, 2019), which involved graduated cylinders to quantify water absorption and soil mass before and after oven drying at 105°C.

### 2.4 Morphological and molecular detection of *Biomphalaria* snails and their trematode infections

*Biomphalaria* snails were collected using scoops and forceps and placed in moist, perforated containers for transport to the tropical and infectious disease laboratory at the Kenya Institute of Primate Research (KIPRE), Nairobi. Initial identification of the snails was performed in the field based on morphological features as outlined by Brown and Kristensen (1993). Snails were then individually exposed to a 60-watt bulb in 24-well plates for two hours to facilitate the shedding of cercariae. The cercariae were identified morphologically using a dissecting microscope, based on standard morphological keys, including forked tail morphology and characteristic swimming behavior (Frandsen and Christensen, 1984). Notably, simple-tailed cercariae were also identified. All snails underwent further molecular analysis where species confirmation was subsequently conducted through molecular analysis targeting the ITS1 gene.

Snail tissue was extracted from the shell with a forceps and macerated from the foot–head region following standard molluscan tissue sampling as described by (Kane et al., 2008). DNA was isolated using the Qiagen DNeasy Blood and Tissue Kit (Qiagen, Germany) with minor protocol modifications. DNA concentration and purity was measured using a Qubit Fluorometer (Thermo Fisher Scientific, USA). PCR targeting the ITS1 region was performed using ETTS2 (5′-TAA CAA GGT TTC CGT AGG TGA A-3′) and ETTS17 (5’-CGA GCC GGA TGA TCC ACC GC-3′) primers to simultaneously detect snail and *S. mansoni* DNA Bakuza et al. (2017). ITS1 positive samples were further analyzed using SM-IMRS primers targeting the 18S rDNA region: forward 5′-CGGTGAAACCGCGAATGGCTC-3′ and reverse 5′-CGCACCCGGTTGGTTCTGTTC-3′, to investigate *S. mansoni* phylogenetic relationships Ally et al. (2024a, b). PCR cycling conditions followed those described by Bakuza et al. (2017) and Ally et al. (2024a, b) respectively. PCR products were confirmed on 1% agarose gel: 600 bp for snail DNA and 500 bp for *S. mansoni* using ITS1 primers, and 151 bp for *S. mansoni* using SM-IMRS primers. Positive DNA controls for all molecular assays were prepared as follows: stool samples as described by Kanyi et al. (2024) were collected from laboratory-maintained baboons (*Papio anubis*), that had been experimentally infected with *S. mansoni*. DNA was then extracted using the QIAamp Fast DNA Stool Mini Kit (Qiagen, Germany), following the manufacturer’s protocol.

### 2.5 Sanger sequencing and phylogenetic analysis

PCR-positive samples including snail DNA, cercarial DNA from field-collected snails, and stool-derived DNA from experimentally infected baboons from KIPRE were submitted to the International Livestock Research Institute (ILRI), Nairobi, for bi-directional Sanger sequencing using the Applied Biosystems 3500XL Genetic Analyzer (Thermofisher, USA). Sequence trace data were inspected in Chromas Pro v2 to confirm accurate peak-to-base alignment prior to trimming. Sequences that failed to meet quality standards were excluded from downstream analyses. Consensus sequences were extracted and compared to GenBank entries using BLASTN sequence homology searches. Multiple sequence alignment including reference sequences retrieved from NCBI was performed using MUSCLE in MEGA 11, and phylogenetic trees were constructed using the Maximum Likelihood method with the Tamura-Nei model. Tree robustness was assessed through bootstrap analysis (1,000 replicates), and final visualization was done in R using the ape package (Paradis et al., 2004).

## 3 Results

### 3.1 Association between snail infectivity and environmental parameters

**Figure 2:**
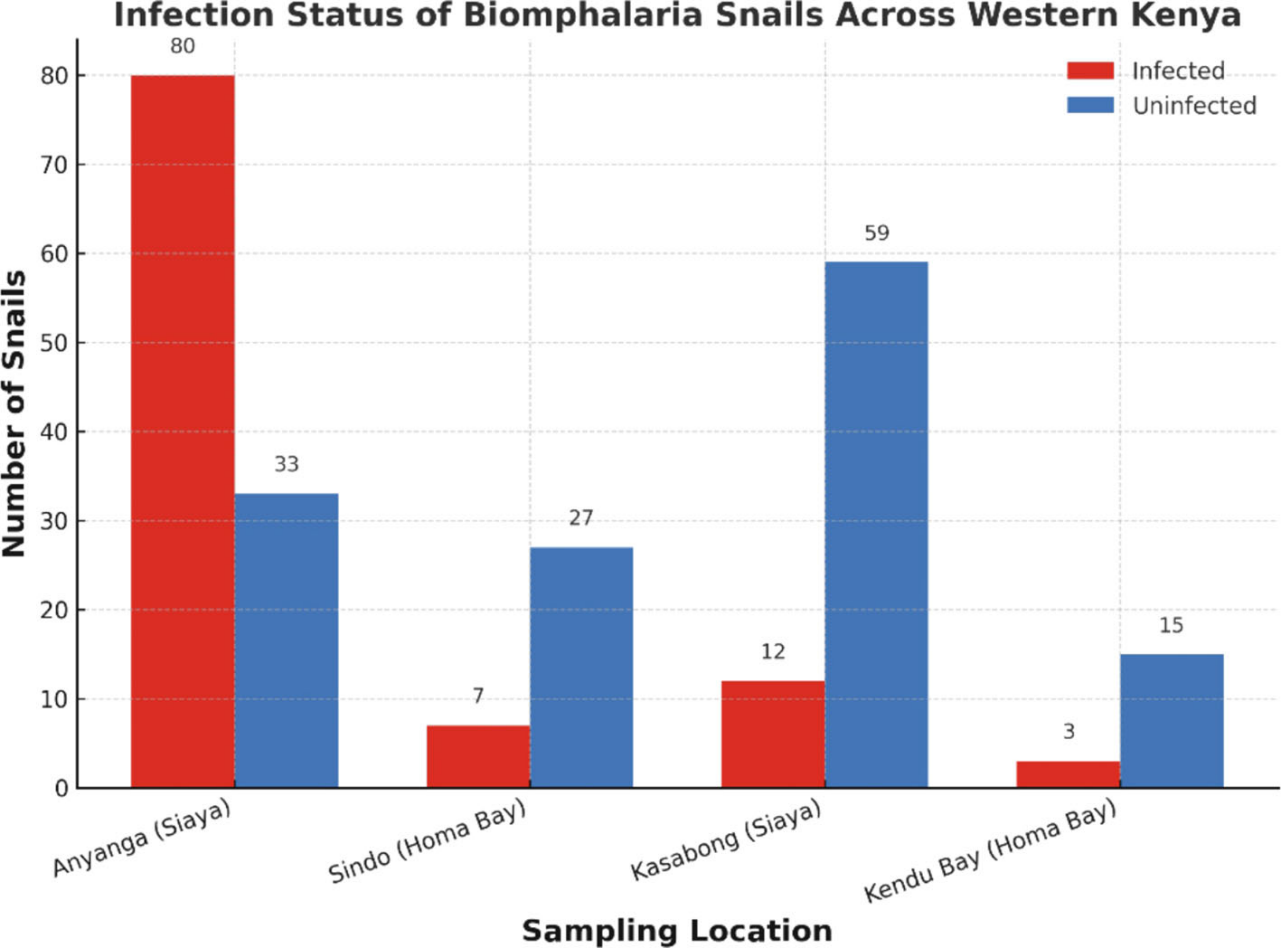
Spatial variation in infection prevalence among *Biomphalaria* snails in western Kenya. The number of infected and uninfected snails collected from Anyanga (Siaya), Kasabong (Siaya), Sindo (Homa Bay), and Kendu Bay (Homa Bay) is shown. Infection prevalence was highest in Anyanga (70.8%, 80/113), followed by Sindo (20.6%, 7/34), Kasabong (16.9%, 12/71), and Kendu Bay (16.7%, 3/18). A Pearson’s chi-squared test revealed a significant association between site and infection status (χ² = 67.325, df = 3, p < 1.596 × 10^-14^), indicating spatial heterogeneity in transmission risk across the study area.

**Figure 3:**
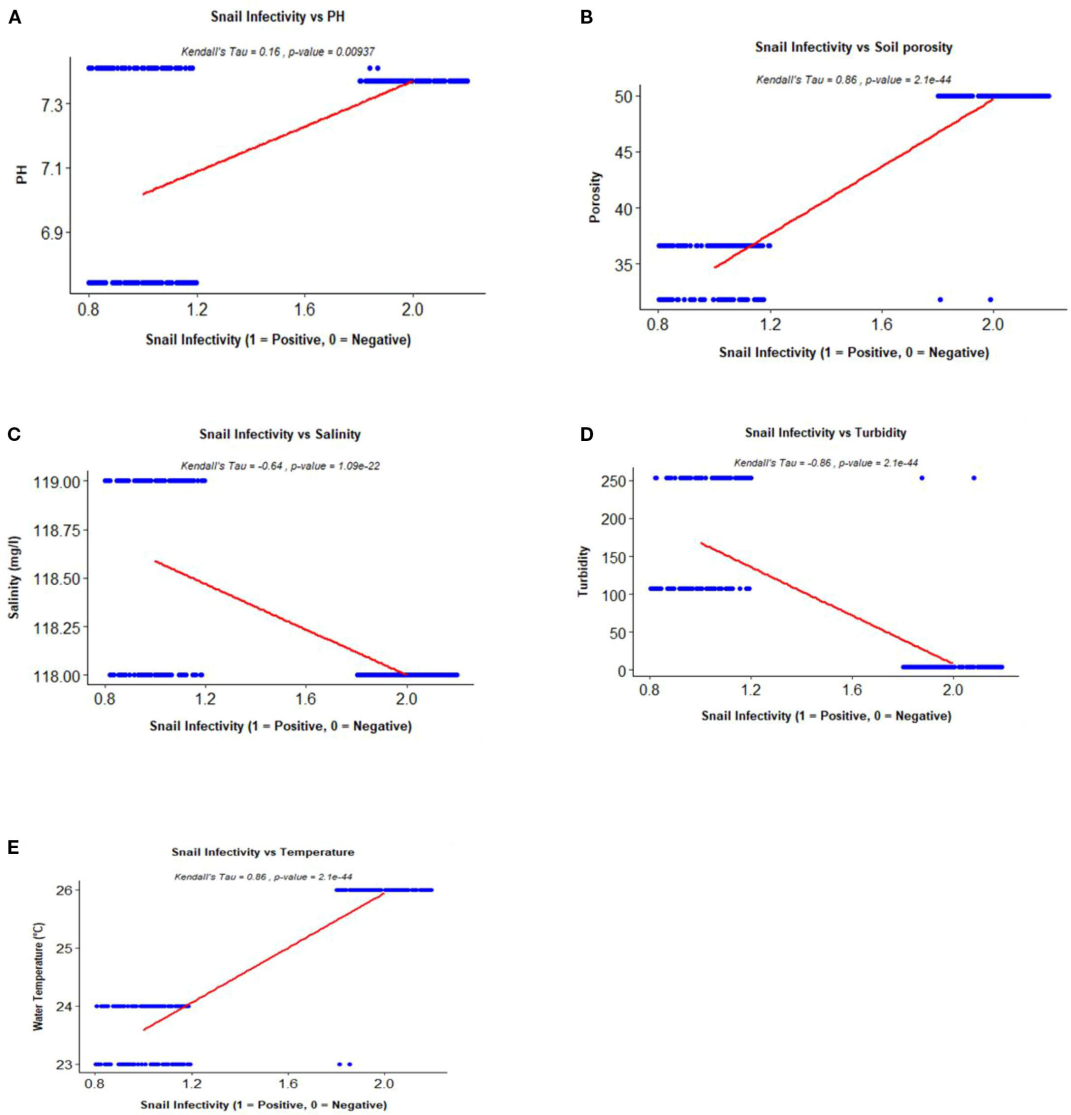
Kendall’s Tau correlation plots (with p-values) illustrating the relationships between infectivity in *Biomphalaria* snails and key environmental variables: pH **(A)**, soil porosity **(B)**, salinity **(C)**, turbidity **(D)**, and temperature **(E)**.

A total of 272 *Biomphalaria* snails were sampled across all study sites, of which 246 were morphologically identified as *B. pfeifferi* and 26 as *B. sudanica* (**Table 1**). Cercarial shedding revealed that 116 snails were infected with trematodes, corresponding to an overall infectivity prevalence of 42.6%. Of these, 102 infected snails originated from sites around Lake Victoria, while 14 were from the Mwea Irrigation Scheme. *Biomphalaria pfeifferi* exhibited marked variation in infection prevalence across sampling sites in western Kenya. The highest prevalence was observed at Anyanga Beach, Siaya County (70.8%, 80/113). Intermediate infection levels occurred at Sindo Rangwena in Homa Bay County (20.6%, 7/34) and Kasabong in Siaya County (16.9%, 12/71), while the lowest prevalence was recorded at Kendu Bay, Homa Bay County (16.7%, 3/18). A Pearson’s chi-squared test revealed a highly significant association between site and infection status (χ² = 67.33, df = 3, p < 0.001), indicating strong spatial heterogeneity in parasite transmission risk across the region (**Figure 2**). Infection levels exhibited a significant positive correlation with both water temperature and soil porosity (**Figures 3B, E**, respectively). Additionally, a weak but positive association was observed with pH (**Figure 3A**), suggesting that pH may have a secondary influence on parasite transmission dynamics. In contrast, salinity (**Figure 3C**) and turbidity (**Figure 3D**) showed negative correlations with infectivity, implying that these parameters may act as ecological constraints on transmission.

**Table 1:** Distribution of *Biomphalaria* snail species across sampling sites in the Lake Victoria basin and Mwea Irrigation Scheme.

Sites	Snail species	
	*Biomphalaria sudanica*	*Biomphalaria pfeifferi*
Anyanga, Siaya (Lake Victoria basin)	5	108
Kasabong’, Siaya (Lake Victoria basin)	13	58
Sindo Rangwena, Homabay (Lake Victoria basin)	8	26
Kendu bay, Homabay (Lake Victoria basin)	0	18
Murinduko, Mwea Irrigation Scheme	0	9
Thiba, Mwea Irrigation Scheme	0	12
Togonye, Mwea Irrigation Scheme	0	8
Kianganga, Mwea Irrigation Scheme	0	7
**Total**	**26**	**246**
**Abundance**	**9.5%**	**90.4%**

Bold values indicate the proportional abundance (%) of each *Biomphalaria* species calculated from the total snails sampled.

### 3.2 Molecular detection of *Biomphalaria* snails and their trematodes

Out of 272 snail-derived samples screened using ITS1 primers, 113 yielded PCR amplicons. Representative PCR-positive samples were selected by site for sequencing to validate species identification and assess phylogenetic relationships. Eight out of 15 samples were successfully sequenced and BLAST analysis revealed that the 600 bp fragment corresponded to *Biomphalaria pfeifferi* found in Mwea Irrigation Scheme and Lake Victoria sites, including Homa Bay and Siaya and the 500 bp band as *S. mansoni* in laboratory-maintained samples at KIPRE and in Thiba (Mwea Irrigation Scheme). The Sequences were submitted to GenBank with accession numbers (PX580465-PX580472).

Following ITS1 screening, the 113 samples that tested positive were further analyzed using SM-IMRS primers targeting the 18S rDNA region. Ninety-seven samples were PCR positive and 22 representative samples were selected for sequencing and downstream analysis. Forty-four high-quality sequences were obtained from cercariae shed by *Biomphalaria* snails collected around the Lake Victoria basin. Both forward and reverse reads were successfully generated and retained, while samples that did not yield reliable trace data were excluded from further analysis. BLAST analysis revealed highest similarity (≥98%) to *Zygocotyle lunata* reference sequences in GenBank accession number (MH915581.1, KM538164.1), confirming species-level identity. No significant matches to other *amphistome* genera were observed and in consideration of the snail species collected in the field. The sequences were submitted to GenBank with accession numbers (PX448099-PX448120).

### 3.3 Phylogenetic analysis

**Figure 4:**
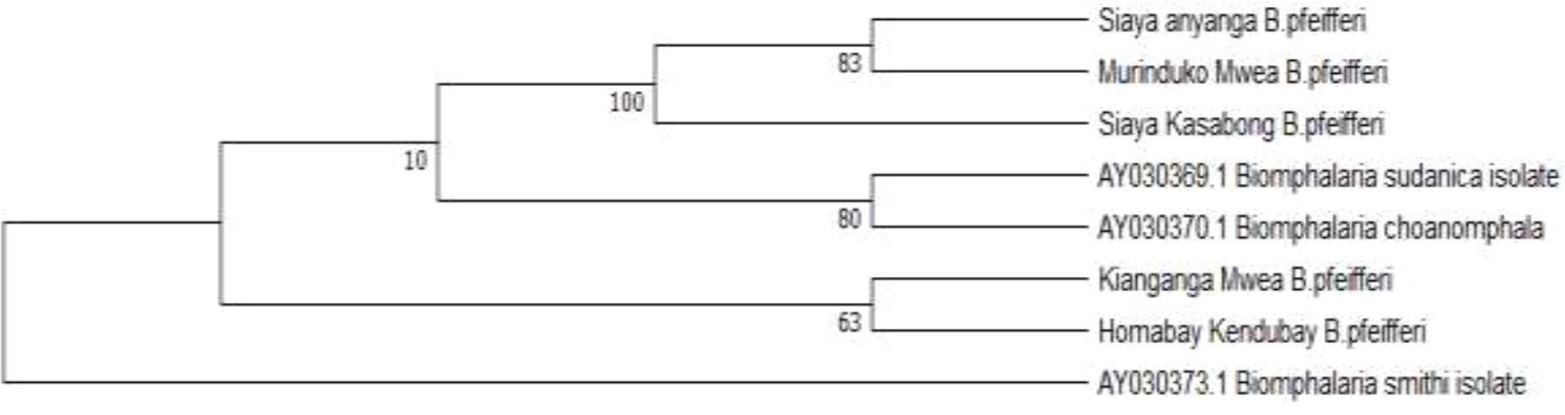
Phylogenetic tree illustrating Kenyan *Biomphalaria pfeifferi* isolates from Siaya (Anyanga, Kasabong) and Mwea (Murinduko, Kianganga) cluster together, confirming species identity. Homabay Kendubay isolates group near reference sequences of *B*. *sudanica* and *B*. *choanomphala*, while *B*. *smithi* serves as an outgroup.

Maximum Likelihood analysis of ITS1 sequences (600 bp) grouped all field *Biomphalaria pfeifferi* isolates into a monophyletic clade, confirming species identity. Bootstrap support was strong for samples collected in Kasabong in Siaya and Murinduko in Mwea Irrigation Scheme (100) and moderate for those in Anyanga, Siaya (83), indicating close phylogenetic relationships among Lake Victoria basin and Mwea Irrigation Scheme populations. Kendubay, Homabay and Kianganga, Mwea Irrigation Scheme isolates clustered near reference sequences of *B. sudanica* and *B. choanomphala* (bootstrap = 63–80), suggesting phylogenetic proximity among East African *Biomphalaria* species. *Biomphalaria smithi* served as an outgroup, reinforcing species-level separation (**Figure 4**).

**Figure 5:**
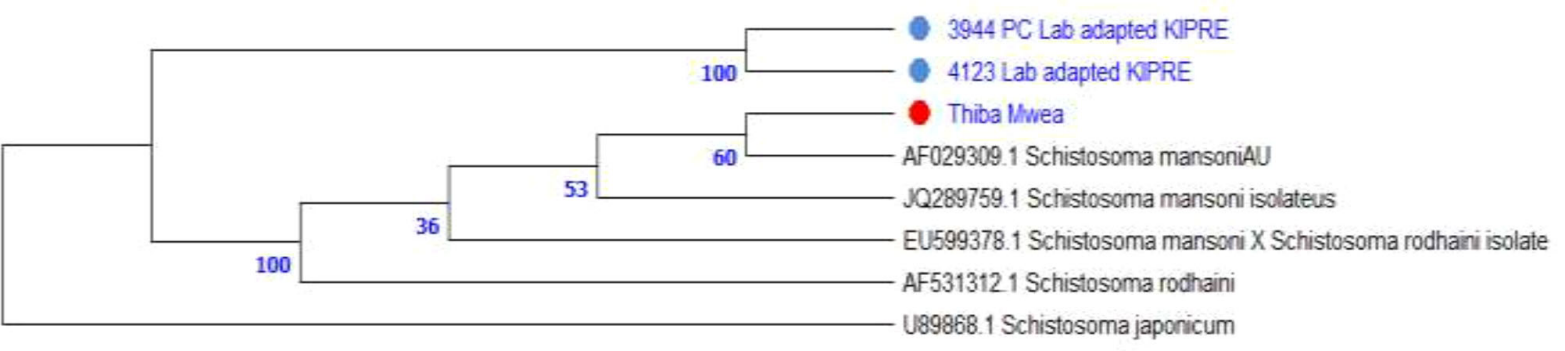
Maximum Likelihood phylogenetic tree of ITS1 sequences (~500 bp) shows *S. mansoni* isolates (colored dots) from Thiba in Mwea Irrigation Scheme, Kirinyaga County and lab adapted samples from KIPRE clustering within the *S. mansoni* clade, while remaining distinct from *S. rodhaini* and *S. japonicum* (outgroup).

Phylogenetic analysis of ITS1 (~500 bp) placed all field isolates within the *S. mansoni* clade with strong bootstrap support (=100 for lab-adapted strains). The Thiba, Mwea Irrigation Scheme isolate is grouped closely with KIPRE strains, confirming species identity. All local isolates were distinct from *S. rodhaini* and *S. japonicum*, indicating no cross-species contamination (**Figure 5**).

**Figure 6:**
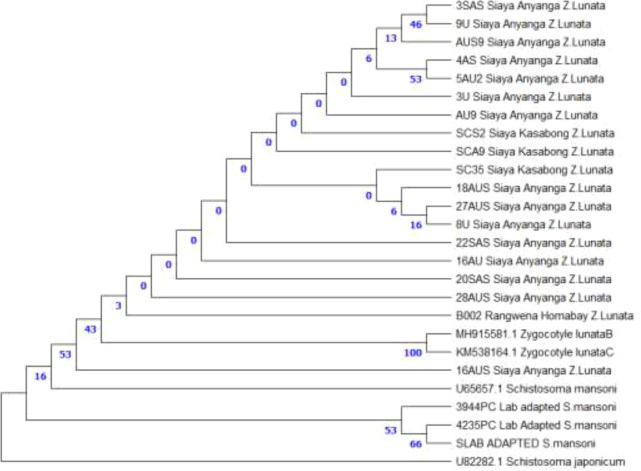
Maximum Likelihood analysis of 18S sequences placed all isolates collected in areas around Lake Victoria in a monophyletic *Zygocotyle lunata* clade with reference sequences (MH915581.1, KM538164.1). Bootstrap support for subclades among those isolates was low (0–53), The node joining these isolates and reference sequences showed strong support (bootstrap = 100), confirming species identity. *Schistosoma mansoni* formed a distinct sister clade (bootstrap = 66), with *S. japonicum* as the outgroup, indicating clear separation between *amphistomes* and *schistosomes*.

Analyzing the Maximum Likelihood for the 18S rDNA sequences located all samples collected around Lake Victoria basin (Anyanga and Kasabong’ in Siaya county and Rangwena in Homabay county) in a well-supported monophyletic clade of *Zygocotyle lunata* next to the reference sequences (MH915581.1, KM538164.1). Support for clades among the Lake region isolates was low (0-53), however, the node that united the Lake region sequences to the reference sequences was well-supported (bootstrap = 100), confirming the species identity. *S. mansoni* isolates formed a distinct sister clade (bootstrap = 66), clearly separating *amphistomes* from *schistosomes*, with *S. japonicum* as an outgroup (**Figure 6**).

## 4 Discussion

Our findings reinforce that schistosomiasis remains a persistent public health concern in regions of Kenya historically classified as endemic, including areas surrounding the Lake Victoria basin and the Mwea Irrigation Scheme (Masaku et al., 2020; Ogongo et al., 2022) as evidenced by presence of documented intermediate hosts responsible for transmission (**Figure 4**). In this study, we assessed how environmental parameters of soil and water including water temperature, soil porosity, water turbidity, water pH and water salinity correlated with trematode infection rates in *Biomphalaria* snails collected from different locations in the Mwea Irrigation Scheme and areas around the Lake Victoria basin (**Figure 3**). This study also examined the use of molecular techniques to detect *schistosome* infections in *Biomphalaria* snail intermediate hosts, along with phylogenetic characterization of both *Biomphalaria* snails and parasites they harbor: *schistosomes* and *amphistomes*.

### 4.1 Environmental factors are correlated with snail infectivity

Water turbidity was found to have a negative correlation with parasites infectivity in *Biomphalaria* snails suggesting that increased water turbidity may hinder parasite-host interactions. This could be attributed to various anthropogenic activities, such as animal watering, vehicle washing, and other disturbances, particularly observed at a collection site in Kasabong, Siaya. These activities contribute to elevated suspended particulate matter, which can interfere with parasite transmission. Our findings are consistent with previous studies, including Upatham, (1972a) and Olkeba et al. (2020), which demonstrated that miracidia were unable to infect snails at 500 ppm turbidity when the water became thick and muddy. Further research supports this, showing that *Biomphalaria* snails prefer clear, low-turbidity waters, while high turbidity can disrupt miracidia movement, reduce light penetration, and interfere with chemical signaling necessary for host detection Akande et al. (2013). Recent research indicates that increased turbidity has been linked to lower *Biomphalaria* population densities and reduced egg development, further impacting transmission dynamics (Hailegebriel et al., 2022; Sokouri et al., 2024; Rollinson et al., 2011; Odongo-Aginya and Eshita, 2008). Our findings suggest that high water turbidity, particularly in human-disturbed habitats, may act as a natural barrier to bilharzia transmission by reducing the efficiency of miracidia in locating and infecting *Biomphalaria* snails. This was evident in areas such as Kasabong in Siaya and Sindo Rangwena in Homa Bay, where infection rates were low whereas anthropogenic activities were high. Hailegebriel et al. (2022) corroborates with our findings where high turbidity levels potentially hindered miracidia’s ability to infect *Biomphalaria* snails, resulting in a decreased cercarial output.

Water salinity was found to have a negative correlation with parasites infectivity in *Biomphalaria* snails. There was a slight difference in water salinity between three sites, Anyanga in Siaya with a water salinity of 118 ppm, Kasabong in Siaya and Sindo Rangwena in Homa-Bay with a water salinity of 119 ppm yet the difference in *schistosome* infection rates was significant with Anyanga in Siaya showing a high level of *schistosome* infection rates while Kasabong in Siaya and Rangwena in Homa-Bay showing lower *schistosome* infection rates. Previous research has demonstrated that salinity levels exceeding 1,200 ppm significantly impair miracidia survival and snail infection success Upatham (1972b). Our findings suggest that even at much lower salinity levels, transmission may still be modulated. This observation is supported by Campbell et al. (2025), who found that moderate salinity reduces *B. pfeifferi* reproduction and survival; Erkano (2021), who reported salinity as a limiting factor for snail distribution in Ethiopian freshwater bodies; and Sokouri et al. (2024), who identified salinity as negatively correlated with snail infectivity in Côte d’Ivoire. These studies collectively support our findings that even relatively low salinity levels may influence *schistosome* transmission dynamics.

Environmental conditions, particularly ambient temperature, play a crucial role in shaping host–parasite interactions and influencing disease dynamics (Leicht and Seppälä, 2014). Previous studies have shown that increased temperatures significantly impact *schistosomes* and their interactions with hosts, especially in Sub-Saharan Africa (Barbosa et al., 2016; Woolhouse and Chandiwana, 1990). Temperature influences the maturation, survival, dispersion, and productivity of intermediate host snails, thereby altering transmission dynamics (McCreesh and Booth, 2014; McCreesh et al., 2015). The free-living stages of *Schistosoma* are also temperature-dependent; cercarial production occurs between 15–31°C, which increases host metabolism and output (Poulin and Mouritsen, 2006; Mangal et al., 2008). Our study corroborates these findings where infection rates varied with temperature, peaking at 26°C in Anyanga, Siaya, moderate at 24°C in Kasabong, Siaya and low at 23°C in Sindo Rangwena, Homabay. This finding concurs with Upatham (1972b), who found infection increased from 14.3% at 16°C to 71.4% at 34°C, then declined to 10% at 40°C. Rowel et al. (2015) also reported a positive correlation between 27–28.7°C and intramolluscan survival. Recent Kenyan studies further support this, Magero et al. (2025) recorded a 22.5% *S. mansoni* prevalence in *Biomphalaria pfeifferi* around Lake Victoria has been shown to vary with environmental factors such as water temperature, and shedding rates of 0.67–0.86% have been reported in *B. sudanica* at Mbita in association with local environmental conditions and Aslan et al. (2024) estimated the thermal optimum for transmission at 23.1–27.3°C. These findings underscore temperature as a critical driver of parasite transmission dynamics (Odero et al., 2024).

Snail populations and their susceptibility to parasite infection can be indirectly influenced by soil porosity, which affects water retention, infiltration, and microbial activity. Although research specifically on soil porosity is limited, studies on substrate types emphasize its importance in shaping environmental conditions that drive schistosomiasis transmission dynamics. Kristensen et al. (2001) identified soil porosity as a key factor affecting snail distribution, survival, and vulnerability to infection. In our study, we observed a positive correlation between parasite infection rates in *Biomphalaria* snails and soil porosity. Anyanga (Siaya) recorded the highest porosity (50.02%), followed by Kasabong (Siaya) (36.64%) and Rangwena, Homa Bay (31.79%). Sites with higher porosity corresponded to higher infection rates, suggesting that areas with increased soil porosity may provide more favorable conditions for snail proliferation and parasite transmission. High soil porosity is often associated with greater water retention and more stable aquatic environments, which are critical for snail survival and reproduction (Mahya and Singh, 2022; Pires et al., 2023). These conditions not only allow snail populations to persist but also increase infection risk by prolonging exposure to parasite larvae (Kalinda et al., 2018). Consequently, soil porosity may be a critical ecological determinant of schistosomiasis hotspots and warrants further investigation. Our findings, which revealed higher infection rates in areas with greater porosity, underscore its potential role in shaping transmission dynamics.

Other ecological factors, such as snail population density, competition between snails and competition between parasites potentially played a role in influencing infection dynamics. Higher snail densities observed in Anyanga (Siaya) could have increased the probability of miracidia encountering a suitable host, leading to greater infection rates. In contrast, lower snail densities or competition with other snail species in Kasabong (Siaya) and Homa- Bay may have reduced parasites’ transmission efficiency. In addition, climatic factors could have contributed to regional differences in infection rates. Increased water flow due to rainfall or human activity could have diluted miracidia concentrations, reducing the likelihood of successful infections.

### 4.2 Phylogenetic resolution of snail hosts and *schistosome* isolates using ITS1 sequences

Our findings confirm the presence of *B. pfeifferi* along the shores of Lake Victoria in Siaya (Mutuku et al., 2021; Onyango et al., 2022) and is the dominant species in the Mwea Irrigation Scheme (Ngigi et al., 2019; Magero et al., 2025). This was verified through morphological and molecular analyses. Phylogenetic analysis of ITS1 sequences (600bp) resolved field *Biomphalaria* isolates into a well-supported monophyletic clade of *B. pfeifferi*, confirming species identity consistent with previous studies that demonstrated the utility of ITS markers for species-level resolution in African *Biomphalaria* (DeJong et al., 2001; Zhang et al., 2019). Subclades linking Kasabong (Siaya) and Murinduko (Mwea Irrigation Scheme) exhibited strong bootstrap support (100), while Anyanga (Siaya) grouped moderately (83), indicating close genetic relationships among Lake Victoria basin and Mwea Irrigation Scheme populations. Kendubay (Homabay) and Kianganga (Mwea Irrigation Scheme) clustered near reference sequences of *B. sudanica* and *B. choanomphala* (bootstrap = 63–80), suggesting phylogenetic proximity among East African *Biomphalaria* sp*ecies* as reported in earlier phylogenetic surveys. The placement of *B. smithi* as an outgroup reinforced species-level separation (**Figure 4**). The identified phylogenetic clustering has important implications for schistosomiasis mitigation.

Maximum Likelihood analysis of ITS1 sequences (500bp) placed field isolates within the *Schistosoma mansoni* clade, supported by strong bootstrap values. The Thiba, Mwea isolate is grouped closely with laboratory-adapted strains from KIPRE (bootstrap = 100), confirming species identity. Additional nodes showed moderate support (53 and 60), indicating genetic similarity among field and reference isolates. *Schistosoma rodhaini* formed a separate lineage (bootstrap = 36), and *S. japonicum* served as an outgroup, reinforcing phylogenetic separation between African *schistosomes* and other species. These findings validate the use of ITS1 for species-level resolution and confirm the phylogeny of field isolates within the *S. mansoni* clade (**Figure 5**). Accurate identification of snail hosts and parasite lineages allows for targeted interventions, such as localized molluscicide and predictive risk mapping. Furthermore, combining phylogenetic data with environmental factors like temperature, pH, and soil porosity can help enhance transmission models and optimize resource allocation in endemic locations.

### 4.3 Molecular identification and phylogenetic characterization of *amphistome* trematodes in *Biomphalaria* snails

*Biomphalaria* species have been reported to harbor several parasites, including members of the superfamilies *Clinostomoidea, Diplostomoidea, Echinostomatoidea, Paramphistomoidea*, and *Pronocephaloidea*, with studies revealing aspects of antagonism and competition among these digenean parasites (Esteban et al., 2011). The interactions between *Biomphalaria* species and non-*schistosome* trematodes are essential for understanding population and transmission dynamics, larval development, and the molecular characteristics of snail–trematode associations. These studies also shed light on the ecological impact of trematodes in animal hosts and provide insights into developing biological strategies to control schistosomiasis (Esteban et al., 2011; Laidemitt et al., 2019; Lu et al., 2024).

In our study, molecular techniques confirmed the presence of *Zygocotyle lunata* in the African *Biomphalaria* species, particularly *B. pfeifferi*, from Siaya and Homabay County. There were three lines of evidence for this identification: The first was in the context of the hosts, in which the cercariae were shed from *B. pfeifferi* and *B. sudanica*. The second was the morphology in which the cercariae had features of the *amphistome* type, which was consistent and similar to *Z. lunata* including simple-tailed cercariae (Spatz et al., 2012). The third line of evidence was the molecular data, in which BLAST analysis of the 18S rDNA sequences showed the highest similarity to *Z. lunata* and the other hits were to *amphistome* species that are not found in the *Biomphalaria* snails. The combination of these three lines of evidence supports the identification and classification of this species to *Z. lunata.*

Earlier reports of *Z. lunata* infections within the *Biomphalaria* genus have been confined to South American species, and in particular *B. peregrina* and *B. tenagophila* (Ostrowski de Núñez et al., 2003; Ostrowski de Núñez, 2011), while *B. glabrata* was thought to be refractory. Our phylogenetic analysis show the field isolates clustering in a monophyletic lineage with reference *Z. lunata* sequences, reinforced with high bootstrap values, indicating shared ancestry thereby confirming species level identity (**Figure 6**). The occurrence of *amphistome* type cercariae in conjunction with fork-tailed *schistosome*-type larvae replicates findings from South America where *Z. lunata* and *Schistosoma mansoni* were found sharing a snail host (Spatz et al., 2012). This emphasizes the complexity of trematode communities in African freshwater ecosystems and the high likelihood of multi-parasite assemblages.

*Zygocotyle lunata* is an intestinal fluke of ruminants which results in enteritis and productivity loss, suggesting that the presence of this parasite indicates the potential for parasite transmission to livestock which are likely to graze near the water bodies where the infected snails are found (Pfukenyi and Mukaratirwa, 2018). Our results highlight the urgency of integrated surveillance of the snail-borne parasites to mitigate the impact on public health and livestock production. This aligns with earlier reports identifying cattle, sheep, and other mammals as definitive hosts of *Z. lunata* in Europe and North America (Fried and Huffman, 2009; Achatz et al., 2025), a finding consistent with our field observations near Anyanga (Siaya) where there was presence of anthropogenic activities such as animal watering, specifically for sheep and cattle.

A key aspect to understanding the transmission and evolution of parasites is the phylogenetic relationships of *amphistome* trematodes in snail hosts of medical importance. Well established clades of *Z. lunata* have been reported from South American *Biomphalaria* species and separated from other paramphistomes (Ostrowski de Núñez et al., 2003; Ostrowski de Núñez, 2011). Our study involved the integration of parasite morphological identification, molecular and phylogenetic approaches targeting 18S rDNA where field isolates confirmed species- level identity and clustered within a strongly supported monophyletic clade of *Z. lunata* referenced sequences (**Figure 6**). Parasite presence was corroborated by ecological observations as previously discussed in this study. Our findings help build knowledge of the phylogenetic relationships of *amphistomes*, associations with hosts, and possible epidemiological impacts to livestock in East Africa; it also builds on studies concerning the taxonomy and molecular characteristics of *Z.lunata*.

Although our findings suggest the presence of *Z. lunata* in African *Biomphalaria* species, several limitations need to be noted: identification was based on amplification of the 18S rDNA region, which, although informative, has a lower phylogenetic resolution compared to other markers. More robust strategies targeting ITS2 or mitochondrial COI regions are likely to result in far better discriminatory power and an improved phylogenetic resolution. The results and the inferences we can draw from this are also limited because the study was only performed in two areas Siaya and Homabay counties in Kenya.

## 5 Conclusion

This study demonstrated that environmental factors such as turbidity, temperature and soil porosity significantly influence *Biomphalaria* snail infectivity and, consequently, schistosomiasis transmission in Kenya. High turbidity and salinity were negatively correlated with infection rates, while temperature and soil porosity showed positive associations, likely enhancing parasite-host interactions. The observed spatial heterogeneity in snail infectivity highlights the focal nature of schistosomiasis transmission, consistent with previous findings in East Africa (Odiere et al., 2012; Steinmann et al., 2006). Given the complex interplay of environmental variables in schistosomiasis endemic regions, further research is needed to better understand how subtle ecological changes influence parasite transmission and infection patterns.

Our findings confirm the presence of *S. mansoni* in Thiba within the Mwea Irrigation Scheme, providing critical baseline data to inform targeted schistosomiasis control strategies in this region. Additionally, the detection of *B. pfeifferi*, a known intermediate host, suggests potential local transmission pathways and underscores the need for integrated snail surveillance and management in studied areas.

Our results also suggest presence of *Z. lunata* in African *Biomphalaria* species, specifically *B. pfeifferi* based on molecular and phylogenetic analysis of field isolates collected in areas around Lake Victoria basin, Kenya. Using 18S rDNA sequences amplified with SMIRS primers, BLAST analysis and phylogenetic reconstruction confirmed that Kenyan isolates form a strongly supported monophyletic clade with reference *Z. lunata* sequences, indicating species-level identity. These findings extend the known host range and geographic distribution of *Z. lunata* beyond South America to East Africa.

Given the pathogenic role of *Z. lunata* in ruminants and cattle (Marcilla et al., 2012; Toledo et al., 2015), its presence in local snail populations suggests a potential transmission pathway to livestock, with implications for animal health and productivity. Future studies should focus on prevalence surveys, seasonal dynamics, and experimental infections to clarify the epidemiological significance of this parasite in East Africa.

## Data Availability

The datasets presented in this study can be found in online repositories. The names of the repository/repositories and accession number(s) can be found in the article/supplementary material.
